# Supplementary material for: Transcriptome Analysis Reveals Vimentin-Induced Disruption of Cell–Cell Associations Augments Breast Cancer Cell Migration
Source: Cells. 2022 Dec 13;11(24):4035. doi: 10.3390/cells11244035 (PMC9776984; doi:10.3390/cells11244035)
Supplement: Supplementary file 1 [file cells-11-04035-s001.zip › DEGs and enrichment analysis.zip!/DEGs and enrichment analysis/WT_VIMvsVector GO enrichment/ALL/WT_VIMvsVector_all.GOmf_DAG.pdf]

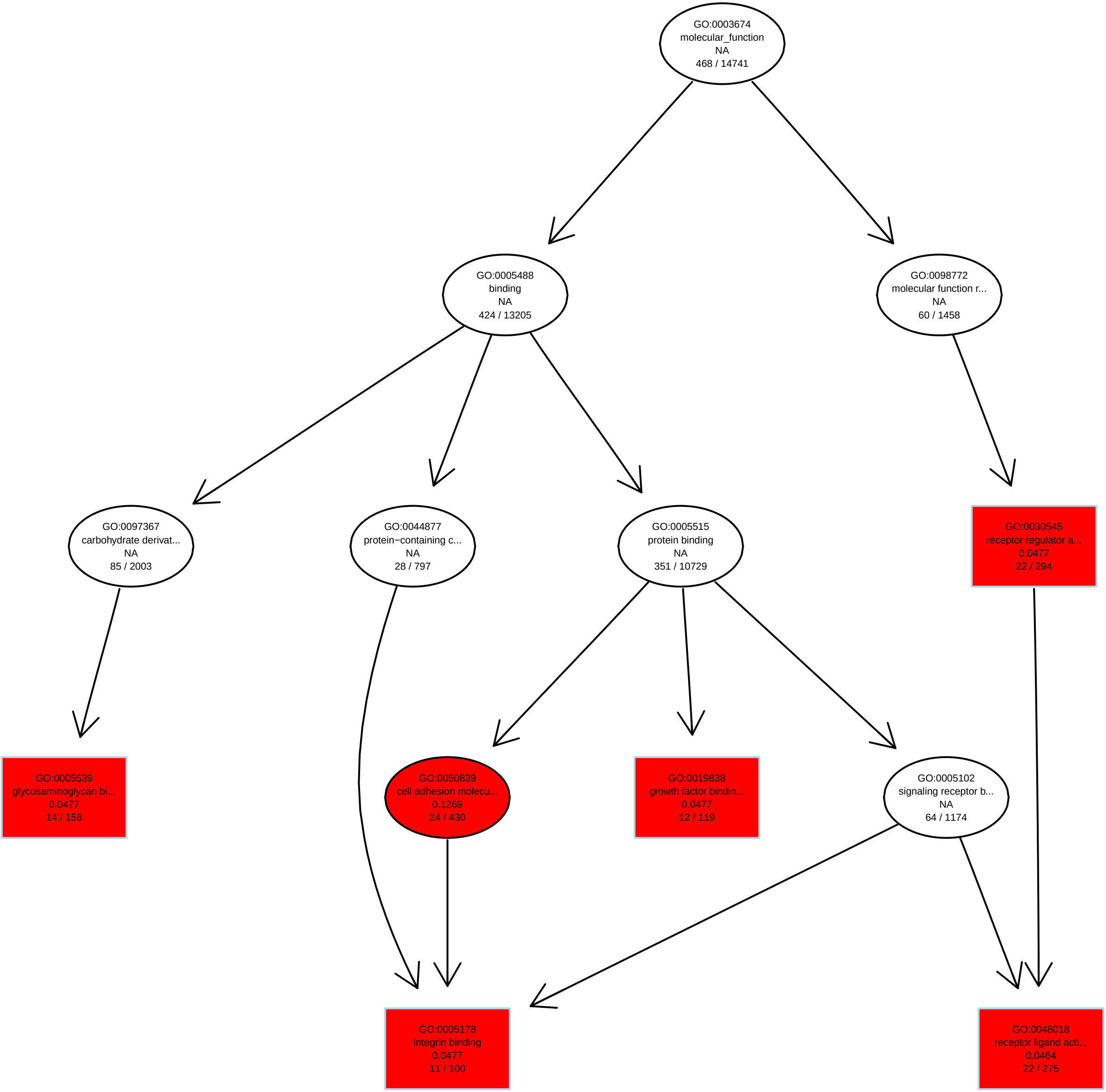

GO:0003674
molecular_function
NA
468 / 14741
GO:0005488
binding
NA
424 / 13205
GO:0098772
molecular function r...
NA
60 / 1458
GO:0097367
carbohydrate derivat...
NA
85 / 2003
GO:0044877
protein−containing c...
NA
28 / 797
GO:0005515
protein binding
NA
351 / 10729
GO:0030545
receptor regulator a...
0.0477
22 / 294
GO:0005539
glycosaminoglycan bi...
0.0477
14 / 156
GO:0050839
cell adhesion molecu...
0.1269
24 / 430
GO:0019838
growth factor bindin...
0.0477
12 / 119
GO:0005102
signaling receptor b...
NA
64 / 1174
GO:0005178
integrin binding
0.0477
11 / 100
GO:0048018
receptor ligand acti...
0.0464
22 / 275
